# Gender and poverty in the United States: Evidence from the Survey of Consumer Finances

**DOI:** 10.1371/journal.pone.0343238

**Published:** 2026-03-11

**Authors:** Patti J. Fisher

**Affiliations:** Virginia Tech, Blacksburg, Virginia, United States of America; Universiti Putra Malaysia, MALAYSIA

## Abstract

This study examines gender differences in poverty determinants among single-headed households in the United States using data from the 2022 Survey of Consumer Finances. Using logistic regression with decomposition methods, we analyzed 1,383 households (833 female-headed, 550 male-headed) to identify factors associated with living below the federal poverty threshold. Results reveal significant differences in poverty determinants between men and women. Working for an employer, self-employment, education level, and age were negatively associated with poverty, while fair health status and income uncertainty were positively associated with poverty risk. The decomposition analysis found a statistically significant coefficient effect (p < 0.0001), indicating that the impact of independent variables on poverty likelihood differs by gender. Employment showed a marginally significant differential effect (p = 0.068), with working for an employer having a smaller protective effect against poverty for women compared to men. However, no constant effect was detected, suggesting gender differences in poverty result from differential responses to specific determinants rather than gender itself. Female-headed households were more likely to have dependent children (38.3% versus 12.7%) and report poorer health status, while male-headed households had higher average net worth. These findings suggest that gender-neutral poverty reduction strategies may be insufficient, and that targeted interventions, such as affordable childcare support to enhance labor market participation among low-income women, are needed to effectively address gender disparities in poverty rates.

## Introduction

The United States is a wealthy nation, but a substantial share of the population is living in poverty [1,2]. According to Fox et al. [3], there has been a growth in deep poverty over the past 20 years. Poverty in the United States is often associated with a lack of housing or substandard living conditions, unsafe neighborhoods, inadequate childcare, under-sourced schools, and hunger [4]. Poverty is associated with a higher risk of mental illness, chronic disease, and shorter life expectancy [5].

U.S. poverty thresholds were defined in the 1960s [1] and the official poverty rate has fluctuated between about 11 and 15 percent since that time. The poverty rate increases when there is an economic downturn and decreases when the economy improves. In 2022, around 37.9 million people were living below the U.S. poverty threshold [6].

Extensive income inequality continues to exist in the United States as of 2020 [7]. The poorest 20% of Americans receive only 3% of all household income. In contrast, the top 20% receive more than 50% of total household income. Empirical research and the media have tended to focus on the top 1% or 5% of the U.S. population in recent years, but less attention has been paid to the lower end of the income distribution. Both scholars and policymakers have called for more attention to households on the lower end of the U.S. income distribution [1].

Women in the United States continue to face higher rates of poverty than men [8]. Women’s access to economic resources and opportunities is more limited and constrained as compared with men [9]. The purpose of this study is to explore gender differences in poverty in the United States.

## Literature review

Despite unprecedented economic and technological progress, a considerable portion of the developed world are unable to maintain a standard of living above the poverty level [10]. The most widely used concept of poverty is based on a lack of economic well-being, with poverty measured by a lack of economic resources for human consumption. Nearly all national and international poverty thresholds represent a variation of economic well-being. In the United States, 37.9 million people were living in poverty in 2022 [6], with an official poverty rate of 11.6% in 2021 [11].

Poverty is commonly defined in the United States as low income [12]. Official poverty statistics in the United States are based on a measure developed in the 1960s [1,6]. The Census Bureau determines a household’s poverty status by using an official poverty measure that compares pre-tax cash income to a specified threshold that is adjusted for family size (Institute for Research on Poverty, 2024).

The official U.S. poverty measure measures the proportion of the population that have total income below the federal poverty threshold given their family size [8]. Income is calculated before taxes and only considers cash income such as wage or salary earnings, pension and retirement income, investment income, Social Security, public assistance (*not* including non-cash benefits), unemployment benefits, alimony, and child support. The 2021 federal poverty threshold for a family of four was $26,500, with a threshold of $12,880 for a one-person household.

Women and non-white women in particular continue to face disproportionate poverty rates [8]. The 2021 official poverty rate for women was 12.6% as compared with 10.5% for men [11]. Nearly half of women in poverty were living in extreme poverty in 2021, which is defined as income at or below 50% of the federal poverty level [8].

For many, falling into poverty is largely dependent on success in the labor market [3]. Low wages are an important factor in poverty, but unemployment and unstable work are the main causes of non-elderly poverty [13]. Unstable employment along with low hours and employment is driving deep poverty for many families [14]. The poverty rate in 2021 was 1.8% for those who worked full-time, year-round, increasing to 12% for those who worked less than full-time, year-round, and 30% for those who did not work at least 1 week [11].

Specific racial and ethnic groups also face a higher risk of poverty [15]. Poverty rates are substantially higher among Native Americans, African Americans, and Hispanics. In 2021, the poverty rate for non-Hispanic white Americans was 8.1%, as compared with 19.5% of Black Americans, 9.3% of Asian Americans, 14.2% of those reporting two or more races, and 17.1% of Hispanics [11].

The poverty rate also varies by education. In 2021, more than one quarter (27.2%) of those without a high school diploma were living in poverty, as compared with 13.2% for those with a high school diploma, 9.2% for those with some college, and 4.1% for those with a bachelor’s degree or higher [11].

## Methods

The 2022 U.S. Survey of Consumer Finances (SCF) is a nationally representative dataset that provides information on household assets, liabilities, and other financial characteristics. The SCF is conducted by the Federal Reserve Board every three years. There are two parts of the SCF sample, first of which is an area-probability sample based on a geographically based random sample. This sample provides information on broadly distributed assets such as homes. The second section is a supplemental list sample, which focuses on wealthy families that hold a large share of less common assets, including non-corporate businesses and tax-exempt bonds. Given that the sample does not follow an equal-probability design, it is important to use weights when interpreting SCF data [16].

Along with the use of weights, the Federal Reserve Board uses multiple imputation techniques in the SCF to deal with missing data [17]. This method involves generating five complete datasets or “implicates” [16]. Using implicates can lead to extra variability in the data, so repeated-imputation inference (RII) techniques should be used to more accurately estimate the empirical results [18] . RII techniques allow for more valid inferences and significance tests [19]  and are used for the logistic regression analyses in this study.

The public SCF data set and the codebook used for this study can be downloaded from the Federal Reserve Board’s website (https://www.federalreserve.gov/econres/scfindex.htm). We use this dataset to investigate gender differences in the characteristics associated with having income below the poverty level. We use decomposition methods to better understand differences in poverty rates between men and women in the United States. After the descriptive analyses, a logit model is estimated with the dependent variable equal to 1 if household income falls below the poverty level given the size of the household, and 0 otherwise.

The sample excludes retired households and households with a married or cohabitating couple. We include only single household heads to compare men and women. The sample includes 1,383 households, with 833 of those headed by women and 550 headed by men. We include the following independent variables in the model: net worth; education (less than high school – reference category, high school or GED, an associate’s degree or some college, and college degree); employment status (not employed – reference, working, self-employed); marital status (never married – reference, widowed, divorced); dependent children living in the home; age in years; race (white – reference, black, Hispanic, other); health status (good or excellent – reference; fair, poor); income uncertainty, defined as not having a good idea of income in the next year; and gender. All VIF values in the model are below 5.

To investigate gender differences in poverty we follow Jackson and Lindley’s [20] method for testing for statistical differences between two groups. The method involves estimating a full interaction model and decomposing any statistical difference to better understand the between-group differences. Data from the two groups (men and women) are pooled to estimate the interaction model. The dependent variable is regressed on an intercept term, the set of independent variables, an indicator variable for gender, and a set of interaction variables created by multiplying each independent variable by the gender variable.

Two additional models are estimated for the decomposition: a reduced model that omits the indicator variable and the set of interaction variables and an intermediate model that includes the indicator variable but omits the interaction variables [20]. The interaction and reduced models are compared to determine the joint significance of the indicator variable for gender and the set of interaction variables. If this joint test is significant, there is a procedure for decomposing the total between-group difference into three components: the constant effect, the endowment effect, and the coefficient or response effect.

The endowment effect is the part of the total difference from differences between the two groups in the level of the explanatory variables [20]. The coefficient or response effect measures the difference between the two groups in the response of the dependent variable to changes in the independent variables. To determine whether a coefficient effect exists, the interaction model and the intermediate model are compared. The portion of the total difference that cannot be accounted for by differential responses is the constant effect, and the estimated parameters on the gender indicator variable in the interaction model indicate whether there is a significant constant effect. A significant coefficient effect provides evidence of gender differences in the impact of the independent variables on being in poverty. A significant constant effect provides evidence of gender differences in the probability of being in poverty.

## Results

### Univariate results

The univariate results presented in Table 1 show that households headed by men and have income below the poverty level have significantly higher average net worth as compared with women ($489,310 versus $250,917, respectively). A significantly higher proportion of men are self-employed (14.6% versus 10.0% of women). The marital status of households in poverty differs significantly. About 65.7% of men in the sample are never married as compared with 48.1% of women. Women in the sample are more likely to be widowed (41.9% of women versus 29.5% of men) or divorced (10% of women versus 4.8% of men).

**Table 1:** Univariate results.

Variable	Total Sample (*n = 1,383*)	Women (*n = 833*)	Men (*n = 550*)
Poverty	16.1%	16.9%	14.8%
**Net worth**	Mean: $345,694 (median: $36,270)	Mean: $250,917 (median: $33,950)	Mean: $489,310 (median: $37,770)
Work for employer	68.0%	67.0%	69.3%
**Self-employed**	11.8%	10%	14.6%
Education			
Less than high school (ref)	8.9%	9.4%	8.2%
**High school**	24.1%	21.8%	27.5%
Associates or some college	31.6%	33.4%	28.9%
College degree	35.4%	35.4%	35.3%
Marital status			
**Never married (ref)**	55.1%	48.1%	65.7%
**Widowed**	37.0%	41.9%	29.5%
**Divorced**	7.9%	10.0%	4.8%
**Dependent children**	28.2%	38.3%	12.7%
Health status			
**Good or excellent (ref)**	68.9%	66.1%	73.1%
Fair	26.2%	28.0%	23.5%
**Poor**	5.0%	6.0%	3.5%
**Age**	45 years	46 years	44 years
Household head race			
**White (ref)**	59.4%	55.4%	65.3%
**Black**	22.4%	26.3%	16.6%
Hispanic	11.6%	12.1%	10.7%
Other	4.3%	3.6%	5.4%
Family assistance	58.0%	56.0%	61.0%
**Income uncertainty**	45.2%	47.6%	41.5%

Note: Bold indicates a significant difference between men and women (*p* < 0.05).

A significantly higher proportion of households headed by women have dependent children (38.3% for women-headed households versus 12.7% for men). Health status also differs for men and women in the sample. About 73% of men report being in good or excellent health while 66.1% of women report good or excellent health. A higher proportion of women report being in poor health (6%) as compared with men (3.5%).

The racial distribution of the sample also differs for men and women. About 65% of male-headed households in the sample are white, as compared with about 55% of female-headed households. A higher proportion of female-headed households living in poverty are black (26.3%) as compared with male-headed households (16.6%). Income uncertainty also differs significantly by gender, with 47.6% of women and 41.5% of men experiencing income uncertainty.

### Logit regression results

The logistic regression results reported in Table 2 show that, after adjusting for other variables in the model, fair health versus good/excellent health and income uncertainty are significantly and positively associated with the likelihood of having income below the poverty threshold. In the interacted model, fair health and income uncertainty are no longer significant. Variables with a significant and negative association with being in poverty are working for an employer, self-employment, having at least a high school education, and age. The R^2^ values for the reduced, intermediate, and interacted models were 0.1946, 0.1949, and 0.2038, respectively. The max-rescaled R^2^ values were 0.3283, 0.3288, and 0.3437, respectively.

**Table 2:** Logistic Regression Results.

Variable	Reduced Model	Intermediate Model	Interacted Model
Net worth	−0.024	−0.019	−0.017
Work for employer	−1.788***	−1.817***	−2.454***
Self-employed	−0.854**	−0.866**	−1.221*
High school diploma	−0.906***	−0.999***	−1.113*
Associates or some college	−1.140***	−1.206***	−1.183*
College degree	−1.991***	−1.978***	−1.673**
Widowed	0.045	−0.001	−0.830
Divorced	0.036	0.049	0.043
Dependent children	0.230	0.175	0.415
Fair health	0.550**	0.596**	0.391
Poor health	0.451	0.542	−0.145
Age	−0.046***	−0.049***	−0.052**
Black household head	0.284	0.294	0.190
Hispanic household head	−0.030	0.007	−0.073
Other race/ethnicity	0.500	0.316	−0.422
Family support	−0.350	−0.370	−0.013
Income uncertainty	0.540**	0.515**	0.557
Female head	–	0.102	−0.536
Net worth*female			−0.015
Work*female	–	–	0.947 (p = 0.068)
Self-employed*female	–	–	0.410
High school*female	–	–	0.215
Associates or some college*female	–	–	−0.028
College degree*female	–	–	−0.625
Divorced*female	–	–	1.042
Widowed*female	–	–	−0.012
Fair health*female	–	–	−0.343
Poor health*female	–	–	0.303
Dependent children*female	–	–	0.927
Age*female			0.006
Black household head*female	–	–	0.208
Hispanic household head*female	–	–	0.139
Other race/ethnicity*female	–	–	1.441
Family assistance*female	–	–	−0.527
Income uncertainty*female	–	–	−0.047

Note: *p < 0.05; **p < 0.01; ***p < 0.001.

### Decomposition results

We first compare the reduced model (−2 Log L = 4,325.61, 17 d.f.) to the interaction model (−2 Log L = 4,253.06, 35 d.f.) to test for an endowment effect. The endowment effect is the portion of the total difference accounted for by differences in the level of the explanatory variables between the two groups. The joint test of the gender indicator variable and the set of interaction terms is significantly significant (*p* < 0.0001). This indicates that there are gender differences in the determinants of poverty. The effects of the independent variables included in the model on poverty differ for single men and single women.

Next, we compare the interaction model to the intermediate model (−2 Log L = 4,323.25, 18 d.f.). The results show a statistically significant coefficient effect (*p* < 0.0001). A significant coefficient effect provides evidence of gender differences in the impact of the independent variables on the likelihood of poverty.

The female variable in the interacted model is not significant, which means there is no evidence of a constant effect. This means there is no evidence of gender differences in poverty beyond the factors adjusted for in the model. A statistically significant coefficient effect and insignificant constant effect support the argument that gender differences in poverty are due to gender differences in the individual determinants of poverty, not to gender in and of itself.

No interacted variables in the interaction model are significant at the p < 0.05 level, but the coefficient variable for female*working for an employer is significant at the p < 0.10 level (p = 0.068). Working for an employer is associated with a significantly lower likelihood of having income below the poverty line; however, the effect of working for an employer has a smaller effect for women than men. This could result from a higher proportion of women having dependent children in the household.

## Discussion and conclusions

Higher-income individuals in the United States have an advantage in nearly every area of life, including housing, neighborhoods, schools, and the labor market [1]. Low-income individuals, on the other hand, face compounding disadvantages in these domains. The current results indicate that gender differences in poverty in the United States exist, and more research is needed.

Although the results show that the determinants of living in poverty differ between men and women, the interaction model shows few significant interaction effects. This pattern of weak interaction effects has implications for understanding gender differences in poverty. It suggests that the factors influencing poverty risk, such as education, employment, and health status, operate similarly for men and women. The near-significant interaction between gender and employment suggests that working for an employer reduces poverty risk for both groups, but there are barriers limiting the poverty-reducing benefits of employment for women. This aligns with the descriptive results showing that 38% of female-headed households have dependent children as compared with only 13% of male-headed households. This difference likely constrains women’s ability to fully benefit from employment opportunities.

According to previous researchers, increasing work rates is one possible way to reduce poverty [21,22]. Providing good-paying jobs is effective at reducing poverty [23]. However, with working for an employer having different relationships with poverty for men and women, additional support for women with dependent children is likely needed. In addition, more exploration of the relationship between working for an employer and the likelihood of poverty is needed.

Lack of affordable and accessible childcare is a problem in the United States, and women are disproportionately affected. Women with dependent children face constraints that can limit their labor market participation and earnings potential, including reduced hours, difficulties accessing reliable and affordable childcare, career interruptions, and limited job flexibility. U.S. safety nets have been shifting from cash-based programs (e.g., TANF) to in-kind benefits such as SNAP, Medicaid, and energy and housing assistance [24,25]. Providing childcare support for low-income women in the United States could help these households engage more in the labor market and reduce poverty. The 26% percentage point difference in dependent children between male- and female-headed households shows a fundamental structural difference between these households that cannot be addressed through employment alone.

The current results show that gender differences in poverty are explained by differential characteristics, not gender itself. The findings suggest that poverty differences between men and women are a result of differences in their individual circumstances and how those circumstances affect poverty risk, rather than from gender discrimination alone. The results also show that employment has a weaker protective effect for women than men. Working for an employer reduces the risk of poverty for both groups, but the smaller effect for women is likely because women often have dependent children in the household, limiting their labor market participation.

Female-headed households in poverty have lower net worth, are more likely to have dependent children, report poorer health status, and experience higher rates of income uncertainty compared to male-headed households. Women in poverty are more likely to be widowed or divorced, while men are more likely to be never married. Women-headed households also have a higher proportion of Black respondents compared to male-headed households. The results indicate that multiple factors protect against poverty. Working for an employer, self-employment, having at least a high school education, and older age are all associated with lower poverty risk, while fair health and income uncertainty increase poverty risk.

There are several policy implications from the current study results. The current findings show that men and women living in poverty face significantly different circumstances. The regression results show that similar factors protect against and increase poverty risk for both men and women, while the descriptive results show that women are more likely to face multiple disadvantages, including responsibility for dependent children, poorer health, lower net worth, and greater income uncertainty. The absence of significant interaction effects should not be interpreted as evidence that gender differences in poverty are unimportant. Rather, the results suggest that gender should be a consideration in poverty reduction approaches to adequately address differential constraints, such as childcare responsibilities, that limit women’s ability to benefit from strategies such as increased employment. Policies that increase work requirements without addressing childcare barriers are unlikely to reduce gender disparities in poverty. Additional studies on poverty alleviation are needed.
